# Methionine Regulates mTORC1 via the T1R1/T1R3-PLCβ-Ca^2+^-ERK1/2 Signal Transduction Process in C2C12 Cells

**DOI:** 10.3390/ijms17101684

**Published:** 2016-10-11

**Authors:** Yuanfei Zhou, Jiao Ren, Tongxing Song, Jian Peng, Hongkui Wei

**Affiliations:** 1 Department of Animal Nutrition and Feed Science, College of Animal Science and Technology, Huazhong Agriculture University, Wuhan 430070, Hubei, China; zhouyuanfei@mail.hzau.edu.cn (Y.Z.); wrjiaofei008@163.com (J.R.); stx901109@163.com (T.S.); pengjian@mail.hzau.edu.cn (J.P.); 2 The Cooperative Innovation Center for Sustainable Pig Production, Wuhan 430070, Hubei, China

**Keywords:** T1R1/T1R3, mTORC1, Ca^2+^, methionine

## Abstract

The mammalian target of rapamycin complex 1 (mTORC1) integrates amino acid (AA) availability to support protein synthesis and cell growth. Taste receptor type 1 member (T1R) is a G protein-coupled receptor that functions as a direct sensor of extracellular AA availability to regulate mTORC1 through Ca^2+^ stimulation and extracellular signal–regulated kinases 1 and 2 (ERK1/2) activation. However, the roles of specific AAs in T1R1/T1R3-regulated mTORC1 are poorly defined. In this study, T1R1 and T1R3 subunits were expressed in C2C12 myotubes, and l-AA sensing was accomplished by T1R1/T1R3 to activate mTORC1. In response to l-AAs, such as serine (Ser), arginine (Arg), threonine (Thr), alanine (Ala), methionine (Met), glutamine (Gln), and glycine (Gly), Met induced mTORC1 activation and promoted protein synthesis. Met also regulated mTORC1 via T1R1/T1R3-PLCβ-Ca^2+^-ERK1/2 signal transduction. Results revealed a new role for Met-regulated mTORC1 via an AA receptor. Further studies should be performed to determine the role of T1R1/T1R3 in mediating extracellular AA to regulate mTOR signaling and to reveal its mechanism.

## 1. Introduction

The mammalian target of rapamycin (mTOR) is a central metabolic regulator that has been implicated in metabolic diseases and is an important effector of metabolic signaling [1,2]. mTOR forms two distinct structural and functional complexes, namely, mTORC1 and mTORC2. mTORC1 promotes cell growth primarily by phosphorylating p70 ribosomal S6 kinase 1 (S6K1) and eukaryotic initiation factor 4E-binding protein 1 (4E-BP1); thus, protein translation is promoted [1,3,4].

mTORC1 activity is regulated by nutrients, energy levels, and growth factors [5,6,7,8]. For example, amino acids (AA) are necessary to activate mTORC1 with other stimuli, such as growth factors. AA-induced mTORC1 activation also involves several events. Branched-chain AAs, particularly leucine, are potent activators of mTORC1 [9]. In HeLa cells, leucine uptake is controlled by SLC7A5/SLC3A2, a bidirectional AA transporter that effluxes intracellular glutamine in exchange for extracellular leucine [10].

Although the mechanisms by which AA transporters couple to mTORC1 have been described, the involvement of AA receptors in mTORC1 activation has been poorly understood. For example, T1R1/T1R3 is a receptor composed of a heterodimer with taste-specific T1R1 and T1R3 G-protein-coupled receptors; taste receptor type 1 member (T1R) 1/3 can serve as a direct sensor of the fed state and AA availability by promoting intracellular Ca^2+^ concentration and activating extracellular signal–regulated kinases 1 and 2 (ERK1/2) to regulate mTORC1 and autophagy [11]. T1R1/T1R3 regulates ERK1/2 and mTORC1 in MIN6 cells [12]. Although T1R1 and T1R3 combine to function as a broadly-tuned l-AA sensor responding to most of the 20 essential AAs [13], sequence differences in T1R receptors within and between species, such as human and mouse, can significantly influence the selectivity and specificity of AA responses [14]. Heterologous expression studies have revealed that mouse T1R1/T1R3 (mT1R1/mT1R3) is broadly activated by most l-AAs, including serine (Ser), arginine (Arg), threonine (Thr), alanine (Ala), methionine (Met), glutamine (Gln) and glycine (Gly), whereas human T1R1/T1R3 (hT1R1/hT1R3) specifically responds to l-Glu and increases intracellular Ca^2+^ concentration [13,15]. The responses of mT1R1/mT1R3 to acidic AAs are also much weaker than its responses to other AAs [13]. Therefore, T1R1/T1R3 possibly mediates the regulation of mTORC1 with AA specificity. Our study confirmed that T1R1/T1R3 functions as a methionine sensor to activate mTORC1 in C2C12 myotubes.

## 2. Results

### 2.1. Amino Acids Activate ERK1/2 and Mammalian Target of Rapamycin Complex 1 (mTORC1) through Stimulation of Ca^2+^ in C2C12 Myotubes

**Figure 1:**
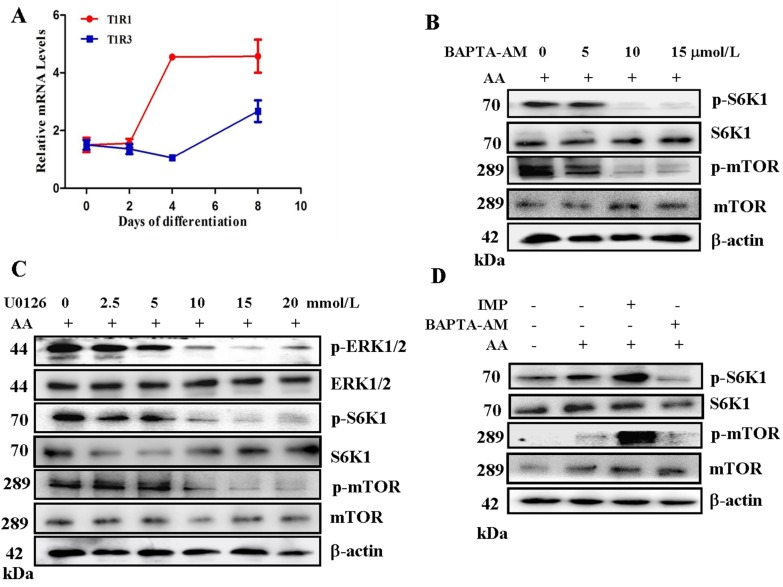
T1R1/T1R3-mediated amino acids regulate ERK1/2 and mTORC1 through stimulation of Ca^2+^ in C2C12 myotubes. RNA was isolated from C2C12 myoblasts of different differentiation times for quantitative polymerase chain reaction (Q-PCR) analysis (**A**); Serum-starved C2C12 cells in Krebs-Ringer’s solution (KRBH) were treated with the indicated concentrations of BAPTA-AM 15 min prior to treatment with AA for 60 min, p-S6K1, p-mTOR protein levels were analyzed by Western blotting, and β-actin was used as a loading control (**B**); C2C12 myoblasts in KRBH were treated with the indicated concentrations of U0126 15 min prior to treatment with AA for 60 min. Immunoblots were performed with the indicated antibodies (**C**); C2C12 cells in KRBH were treated with the 1 mM/L IMP or 10 µM/L BAPTA-AM 15 min prior to treatment with AA for 60 min. Immunoblots were performed with the indicated antibodies (**D**). “+” means to add, “-” means not to add.

T1R1/T1R3 is a direct sensor of AA availability. AAs are sensed directly by T1R1/T1R3, and PLCβ is then activated; calcium influx increases and, thus, stimulates ERK1/2 and ribosomal protein S6 kinase (RSK) to activate mTORC1 [12]. T1R1 and T1R3 are commonly found in mouse tissues, human islets, and cultured cells [12]. C2C12 myoblast is the main cellular model used to investigate protein synthesis in skeletal muscles. We determined whether T1R1/T1R3 may also serve as an AA sensor in C2C12 cells. T1R1 and T1R3 were expressed at various differentiation periods of C2C12 cells (Figure 1A). We examined whether the cell-permeable Ca^2+^ chelator 1,2-bis (2-aminophenoxy) ethane-*N*,*N*,*N*’,*N*’-tetraacetic acid (BAPTA)-acetoxymethyl ester (BAPTA-AM) can inhibit AA-induced S6K1 and mTOR phosphorylation in myotubes. We found that the phosphorylation of mTOR and S6K1 was inhibited by the pre-incubation of BAPTA-AM in a dose-dependent manner (Figure 1B). We also identified whether ERK1/2 contributes to AA-induced mTORC1 activation. U0126, which prevents ERK1/2 activation by blocking MEK1/2, inhibited not only AA-induced ERK1/2 stimulation, but also S6K1 and mTOR phosphorylation. Thus, ERK1/2 participated in mTORC1 regulation in response to AAs (Figure 1C). However, whether this effect depends on T1R1/T1R3 remains unknown. T1R1/T1R3 is also exclusively involved in the synergistic enhancement of umami taste by disodium 5′-inosinate/disodium 5′-guanylate (IMP/GMP) is purinic ribonucleotides, and can strongly potentiate the umami taste intensity. Indeed, the T1R1/T1R3 responses to nearly all l-AAs are significantly enhanced by low IMP doses [13]. In our study, the responses of mTOR and S6K1 phosphorylation to AAs were significantly enhanced by IMP (Figure 1D). These data showed that T1R1/T1R3 can mediate AA-regulated mTORC1 through Ca2^+^ stimulation and ERK1/2 activation in C2C12 cells.

### 2.2. Met Promotes Protein Synthesis

**Figure 2:**
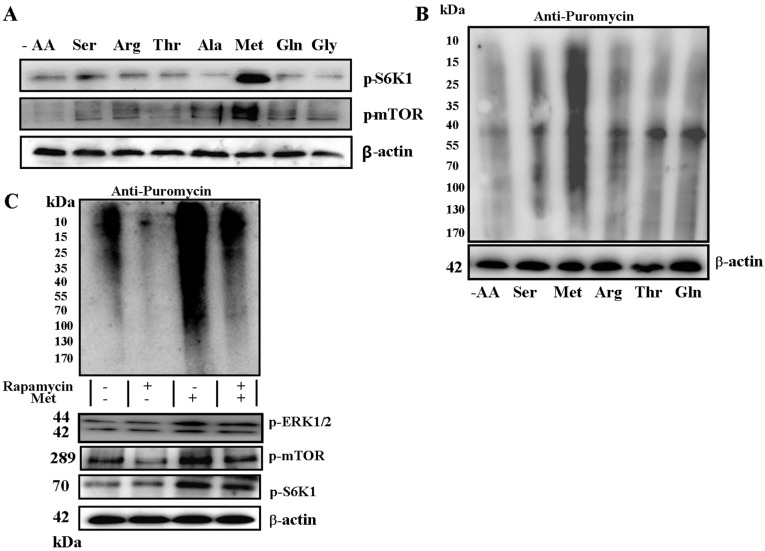
Met activates ERK1/2 and mTORC1 in C2C12 cells. Serum-starved C2C12 cells in KRBH were treated with seven different kinds of AA for 60 min. Immunoblots were performed with the indicated antibodies (**A**); Serum-starved C2C12 cells were stimulated with or without AA (Ser, Met, Arg, Thr, and Gln) and 20 nmol/L Rapamycin for 60 min, and rates of protein synthesis were measured with the SUnSET technique described in Materials and Methods (**B**); Immunoblots were performed with the indicated antibodies (**C**). “+” means to add, “-” means not to add. Results were represented as mean ± SD.

T1R1/T1R3 exhibits species-dependent differences in ligand specificity; for instance, hT1R1/hT1R3 specifically responds to l-Glu and l-Asp, whereas mT1R1/mT1R3 responds more strongly to l-AAs, including l-Ser, l-Thr, l-Ala, l-Gly, l-Met, l-Arg, l-Gln and l-Asn, than it does to l-Glu [14]. However, the specific AA that can be mediated by T1R1/T1R3 to regulate mTOR activity in myotubes remains unknown. In our study, l-Ser, l-Arg, l-Thr, l-Ala, l-Met, l-Gln and l-Gly were used as experimental objects; these AAs can improve the intracellular Ca^2+^ concentration under the action of T1R1/T1R3 [13]. Our results showed that only l-Met significantly promoted mTOR and S6K1 phosphorylation (Figure 2A). We further determined the protein synthesis rates by applying the principles of surface sensing of translation (SUnSET) to develop a nonradioactive method for ex vivo and in vivo measurements of skeletal muscle protein synthesis [16]. We found that l-Met can significantly promote protein synthesis (Figure 2B). Rapamycin treatment inhibited the rate of protein synthesis and the Met-induced increase in ERK1/2, S6K1 and mTOR phosphorylation level (Figure 2C). These results demonstrated that Met can promote protein synthesis.

### 2.3. Phospholipase C (PLC) β-Ca^2+^-ERK1/2 Signal Transduction Process Is Essential for Met-Induced Regulation of mTOR Signaling and Protein Synthesis in C2C12 Myotubes

**Figure 3:**
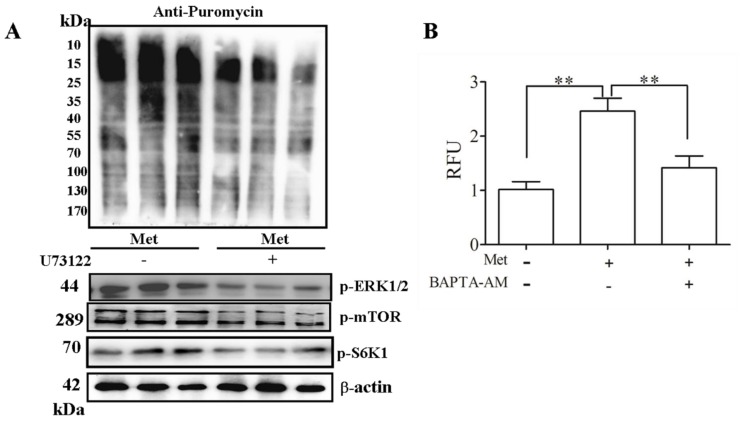

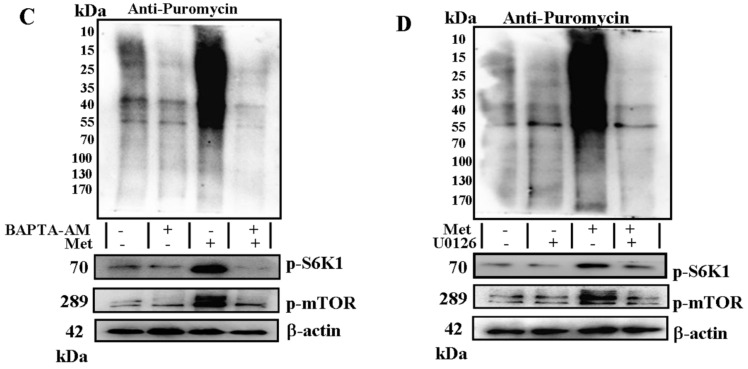
Met regulation of the PLC-Ca^2+^-ERK1/2 signal transduction process was essential for activation of mTOR signaling in C2C12 cells. Serum-starved C2C12 cells in KRBH were treated with 1 µM/L U73122 (**A**); 10 µM/L BAPTA-AM (**C**); and 15 µM/L U0126 (**D**) for 15 min prior to treatment with Met for 60 min, respectively. The cells were loaded with Fluo8-AM as described in Materials and Methods; C2C12 myoblasts were treated with Met for 5 min in the absence or presence of BAPTA-AM (**B**). Immunoblots were performed with the indicated antibodies, and rates of protein synthesis were measured with the SUnSET technique. “+” means to add, “-” means not to add. Results were represented as mean ± SD, ** *p* < 0.01.

In taste cells, signal transduction via T1R1/T1R3 is also mediated by complex Gβγ subunits [17]. As a result, phospholipase C (PLC) β is activated to transduce the umami signal [18]. To examine whether the Met signal from T1R1/T1R3 can activate PLCβ in C2C12 cells, we used U73122, a PLCβ inhibitor. Our results revealed that it could reduce Met-induced mTOR and S6K1 phosphorylation (Figure 3A) and PLCβ could positively regulate Met-induced protein synthesis. The addition of Met evoked a rapid increase in Ca^2+^, which was largely inhibited by pre-incubation with BAPTA-AM (Figure 3B). The protein synthesis rates were also measured via SUnSET, and our results showed that Met could significantly promote protein synthesis in a Ca^2+^ and ERK1/2-dependent manner (Figure 3C,D, respectively). Our results further confirmed that the Met-induced phosphorylation of mTOR and S6K1 was dependent on Ca^2+^ (Figure 3C) and ERK1/2 (Figure 3D). Therefore, Met regulates mTOR signaling and protein synthesis in C2C12 myoblasts through PLCβ-Ca^2+^-ERK1/2 signal transduction.

### 2.4. T1R1/T1R3 Regulated mTORC1 through Ca^2+^ Stimulation and ERK1/2 Activation with Met

**Figure 4:**
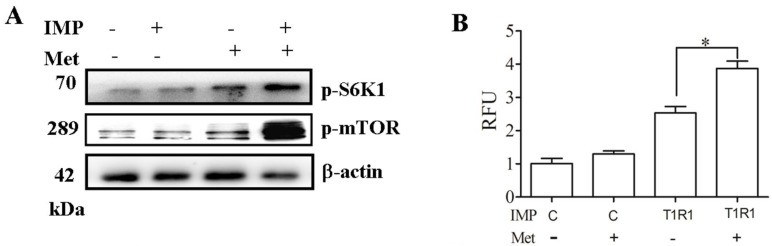

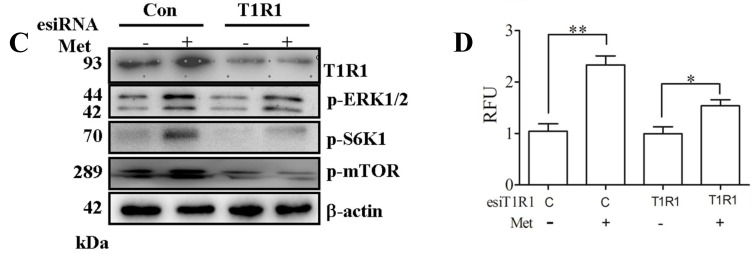
Met activates ERK1/2 and mTORC1 through the T1R1/T1R3 receptor. Serum-starved C2C12 myoblasts in KRBH were treated with 1 mM/L IMP for 15 min prior to treatment with 50 mM Met for 60 min. p-S6K1 and p-mTOR protein levels were analyzed by Western blotting, and β-actin was used as a loading control (**A**); C2C12 myoblasts were treated as in (**A**), loaded with Fluo8-AM, and pretreated with disodium 5′-inosinate (IMP) for 30 min prior to Met stimulation. Data are the mean ± SEM of the relative fluorescence units (RFU) values of three independent experiments (**B**); The control (Con) esiRNA and T1R1 esiRNA (Sigma, St. Louis, MO, USA) were transfected into C2C12 myoblasts in KRBH were stimulated with 50 mM Met for 60 min. T1R1, p-ERK1/2, p-S6K1, and p-mTOR protein levels were analyzed by Western blotting, and β-actin was used as a loading control (**C**). C2C12 myoblasts were treated as in (**C**) and loaded with Fluo8-AM; Data are the mean ± SEM of the RFU values of three independent experiments (**D**). “+” means to add, “-” means not to add. Results were represented as mean ± SD, * *p* < 0.05, ** *p* < 0.01.

Studies have yet to determine whether Met is a ligand of T1R1/T1R3 in mTORC1 signaling. In our study, the effects of IMP on S6K1 and mTORC1 stimulation by Met were examined. IMP promoted S6K1 phosphorylation and mTORC1 activation by Met (Figure 4A). The addition of Met also evoked a rapid increase in Ca^2+^ levels in C2C12 cells, which were significantly increased by pre-incubation with IMP (Figure 4B). The knockdown of T1R1 by esiRNA in C2C12 cells reduced the expression of T1R1, phosphorylation of ERK1/2, Met-induced activation of S6K1 (Figure 4C), and phosphorylation of mTORC1 activation (Figure 4C). The knockdown of T1R1 also significantly decreased the Ca^2+^ levels in C2C12 cells (Figure 4D). These findings showed that T1R1/T1R3 may function as the main or additional Met receptor in mTOR signaling.

## 3. Disscusion

G protein-coupled receptors (GPCRs) participate in sensing nutrients, including glucose and AAs in fungi [19]. In yeast, AA detection by the TOR system involves nutrient transport proteins, and most of these proteins have limited or no ability to transport AAs to the cell interior; instead, they function as membrane receptors [20,21]. In the lingual epithelium, T1R1/T1R3 functions as a broad-spectrum l-AA sensor. In rodents, this receptor responds to most of the 20 essential AA at millimolar concentrations [13]. T1R1 and T1R3 are commonly found in mouse tissues, human islets, and cultured cells [11]. Considering that most cell types require AA-sensing capabilities to coordinate cellular demands with nutrient availability, we determined whether T1R1/T1R3 may also serve as an AA sensor in C2C12 myotubes. We found that T1R1/T1R3 can sense L-AAs to regulate mTORC1 in C2C12 myotubes (Figure 1).

T1R1/T1R3 is activated by most AAs, such as alanine, glutamate, methionine, and glycine, perceived as umami [13]. T1R1/T1R3 exhibits species-dependent differences in ligand specificity; for example, hT1R1/hT1R3 specifically responds to l-Glu, but mT1R1/mT1R3 responds more strongly to l-AAs, including serine, arginine, threonine, alanine, methionine, glutamine, and glycine, than it does to l-Glu [14]. However, we observed that only methionine could activate mTORC1 and promote protein synthesis (Figure 2). The potential importance of methionine as a “nutrient signal” of protein metabolism has been described [22]. In particular, methionine affects mTOR signaling in mammary tissues [9,23]. In summary, this study revealed a novel role of methionine as a regulator of mTOR signaling.

Calcium participates in the regulation of mTORC1/S6K1 activity [24,25]. ERK1/2, through the ERK1/2 substrate RSK, and phosphorylates and inhibits the TSC1/TSC2 complex [26,27]. Thus, ERK1/2 negatively controls mTOR and enhances mTORC1 activity. ERK1/2 may also promote mTORC1 activation by directly phosphorylating Raptor, a subunit of mTORC1 [26,28]. Ca^2+^ and ERK1/2 are also implicated in mTOR activity regulation. T1R1/T1R3 regulates AA-induced mTORC1, and autophagy depends on Ca^2+^ and ERK1/2 [11]. In C2C12 myotubes, methionine-induced S6K1, and mTOR phosphorylation is dependent on Ca^2+^ and ERK1/2. The functional properties of T1R1/T1R3 were further characterized, and the results revealed that IMP, a 5-monophosphate ester functioning as the specific potentiator of T1R1/T1R3 activation [29], significantly enhanced the levels of mTOR activation. Our results also strongly suggested that the response to Met, which was observed in C2C12 myotubes, occurred via T1R1/T1R3 (Figure 3).

The ability of AAs to regulate TOR signaling is functionally conserved across eukaryotes and is essential for mTORC1; AA transporters also function upstream of mTORC1 to allow cells to sense AA availability and stimulate anabolic responses, such as increased translation and growth [30]. AA stimulation, such as l-leucine can improve the transcription and translation of mTORC1 [31,32]. Previous studies focused on the mechanism by which the activity of some AA transporters may modulate the size and composition of the intracellular AA pool and the effects of such an activity [21]. SLC7A5/SLC3A2 is a bidirectional transporter that regulates the simultaneous efflux of l-glutamine out of cells and the transport of l-leucine/EAA into cells, and this exchange is rate-limiting for the activation of mTORC1 by essential AAs and growth factors [10]. l-leucine also improves ribosomal function through mTORC1 signaling to promote cell division and enhance animal development [33]. However, the molecular mechanism of other AAs involved in regulating mTOR signal transduction remains unclear. Specific AAs as sensors on the cell membrane regulate signaling via nutrient-sensitive pathways. For example, methionine regulated mTORC1 via T1R1/T1R3-PLCβ-Ca^2+^-ERK1/2 signal transduction in C2C12 cells. Although glutamine, glycine, and alanine can stimulate Ca^2+^ signaling, these AAs cannot activate mTORC1, possibly because ERK1/2 cannot be activated. Nevertheless, studies have yet to provide evidence supporting that ERK1/2 phosphorylation levels are altered in response to Gln/Gly/Ala stimulation in myoblasts. The protein tyrosine phosphatase SHP-2 is required for leucine-induced activation of S6K1 in skeletal myoblasts [34]. SHP-2 also functions downstream of GPCRs [35]. Therefore, T1R1/T1R3 may be involved in the transduction of SHP-2-mediated nutrient responsiveness.

## 4. Materials and Methods

### 4.1. Cell Culture and Treatment

C2C12 myoblasts were cultured as described previously. C2C12 myoblasts were cultured at 37 °C and 5% CO_2_ in dulbecco’s modified eagle medium (DMEM) (Life Technologies, Ghent, Belgium) containing 10% fetal bovine serum FBS (Life Technologies, Ghent, Belgium), 1 mM sodium pyruvate (Invitrogen, Carlsbad, CA, USA), 5 units/mL penicillin and 50 µg/mL streptomycin (Sigma, St. Louis, MO, USA). Cells were plated on glass coverslips in six-well tissue culture plates and incubated at 37 °C for eight h in growth medium. Then cell differentiation for four days at 37 °C and 5% CO_2_ in DMEM (Life Technologies, Ghent, Belgium) containing 2% horse serum (Life Technologies, Ghent, Belgium), 1 mM sodium pyruvate (Life Technologies, Ghent, Belgium), 5 units/mL penicillin, and 50 µg/mL streptomycin (Sigma, St. Louis, MO, USA). For nutrient starvation before experiments, cells were placed in DMEM without serum for 15 h before incubation in AA-free Krebs-Ringer’s solution (KRBH) supplemented with 0.1% bovine serum albumin (BSA). Indicated cells were stimulated with amino acids (1XAA), 1XAA concentration in the pig plasma, or stimulated with 50 mM/L Met, Leu, Gln, Gly, and Ala, which were incubated in AA-free KRBH buffer for 60 min. Cells were pretreated for 15 min with the T1R1/T1R3 induction AA enhancer (IMP), the active PLCβ inhibitor (U73122), the Ca^2+^ chelator BAPTA-acetoxymethyl ester (BAPTA-AM), and ERK1/2 active inhibitors (U0126) separately before the 60 min AA/Met stimulus period.

### 4.2. Transfection of esiRNA

C2C12 cells were transiently transfected with either esiRNA controls (Sigma-Aldrich; #EHUEGFP, St. Louis, MO, USA) or MISSION esiRNA (Sigma-Aldrich; #EMU049931, St. Louis, MO, USA) directed against T1R1 designed and manufacturered by Sigma-Aldrich with Lipofectamine RNAiMAX (Life Technologies, Ghent, Belgium) following the manufacturer’s protocol and used 48–72 h later.

### 4.3. Quantitative Reverse Transcription-Polymerase Chain Reaction (RT-PCR)

RNA was isolated from C2C12 cells using TRIzol reagent (Life Technologies, Ghent, Belgium) according to the manufacturer’s instructions and followed by DNase digestion using a DNA-free kit (Ambion, Austin, TX, USA) according to the manufacturer’s instructions. Relative mRNA levels of genes were quantified by using a Bio-Rad 129 CFX Connect™ Real-Time PCR Detection System (Bio-Rad, Richmond, CA, USA). The relative expression levels of *T1R1* and *T1R3* mRNA were normalized to β-actin by using the δ-δ method. We used a three-stage PCR program, 94 °C for 5 min, followed by 40 cycles of 94 °C for 30 s, 60 °C for 20 s, 72 °C for 20 s, and a final extension step at 72 °C for 5 min. The sequences of the primers as shown in Table 1.

### 4.4. Nonradioactive Measurements of Protein Synthesis with SUnSET

C2C12 cells were cultured and treated as above, after the AA/Met stimulus for 30 min, then 1 µM puromycin was added to incubate for an additional 30 min. Cells were then harvested and analyzed by Western blotting as described below.

### 4.5. Western Blotting

C2C12 myotubes were washed three times with ice-cold phosphate buffer saline (PBS) and then extracted with a solution kit (Beyotime, Suzhou, China). The protein concentrations were determined using bicinchonininc acid (BCA) protein assay, and proteins were separated by 10% sodium dodecyl sulfate polyacrylamide gel electrophoresis (SDS-PAGE). The separation proteins were transferred to a polyvinylidene difluoride (PVDF) membrane. The membrane was blocked with Tris-buffered saline (TBS) TBS and 0.1% (*v*/*v*) Tween-20 (TTBS) buffer (10 mM Tris-HCl (pH 7.6), 150 mM NaCl, 0.1% Tween) containing 5% skim milk powder for 2 h, and then incubated with the primary antibody for mTOR (#2972; Cell Signaling Technology, Beverly, MA, USA), phospho-mTOR (Ser2448) (#2971; Cell Signaling Technology, Beverly, MA, USA), p-p70S6K (sc-8416, Santa Cruz Biotechnology, Santa Cruz, CA, USA), phospho-p70S6K1 (Thr 389) (sc-11759, Santa Cruz Biotechnology, Santa Cruz, CA, USA), ERK1/2 (#9102, Cell Signaling Technology, Beverly, MA, USA), phospho-ERK1/2 (Thr202/Tyr204) (#9101, Cell Signaling Technology, Beverly, MA, USA), anti-puromycin antibody (MABE343, Billerica, MA, USA), TAS1R1 (ab155143, Abcam Inc., Cambridge, MA, USA) and β-actin (sc-47778, Santa Cruz Biotechnology, Santa Cruz, CA, USA) overnight at 4 °C. Secondary antibodies, anti-mouse, or rabbit IgG-HRP were used to detect primary antibodies. Binding was detected using an enhanced chemiluminescence detection kit (Thermo Fisher Scientific, San Jose, CA, USA) according to the manufacturer’s instructions.

### 4.6. Ca^2+^ Measurement

The intracellular calcium level was measured as described previously [36]. C2C12 cells were cultured at 37 °C and 5% CO_2_ in DMEM containing 10% FBS. When cell fusion was larger than 80%, a 12 h serum-free starvation treatment was applied, and then cells were treated with AA-deprivation DMEM or 2-hydroxyethyl (HEPES)-buffered physiological saline solution (HBSS) for 3 h. Then, the culture medium was replaced and 5 µmol/L of Ca^2+^ sensitive indicator dye Fluo-8 (AAT Bioquest, Sunnyvale, CA, USA) was added for 1 h. After cells were washed twice with HBSS, Fluo-8 fluorescence intensity changes were determined by using a Synergy2 microplate reader (BioTek, Gene Company Limited, Doraville, GA, USA).

### 4.7. Statistics Analysis

All data analysis was performed using GraphPad Prism 5.0 (GraphPad Software Inc., La Jolla, CA, USA). Student’s two-tailed, unpaired *t*-tests were used for all two-group comparisons. The procedure was performed using the SAS System program (SAS Institute Inc., Cary, NC, USA). The statistical significance level was set at *p* < 0.05.

## 5. Conclusions

In summary, T1R1/T1R3 functions as a Met sensor, which transduces signals by increasing intracellular Ca^2+^ concentration and activating ERK1/2 to regulate mTOR signaling in C2C12 myotubes. T1R1/T1R3-PLCβ-Ca^2+^-ERK1/2 signal transduction is necessary to promote Met-regulated mTOR signaling and protein synthesis in C2C12 myotubes.

## Figures and Tables

**Table 1:** Quantitative polymerase chain reaction (Q-PCR) primers.

Gene	Forward Primer	Reverse Primer
*T1R1*	5′-CATCTGGTGATTCTTGAGTG-3′	5′-AGGATACGAAGTGGAGGAG-3′
*T1R3*	5′-CAAGTTCTTCAGCTTCTTCC-3′	5′-GGCGGCCACCCAGTTCCAGC-3′
*β-actin*	5′-GGCACCACACCTTCTACAATG-3′	5′-GGGGTGTTGAAGGTCTCAAAC-3′
